# Implementing ABCD study^Ⓡ^ MRI sequences for multi-site cohort studies: Practical guide to necessary steps, preprocessing methods, and challenges

**DOI:** 10.1016/j.mex.2024.102789

**Published:** 2024-06-01

**Authors:** Wajiha Bano, Elmo Pulli, Lucia Cantonas, Aino Sorsa, Jarmo Hämäläinen, Hasse Karlsson, Linnea Karlsson, Ekaterina Saukko, Teija Sainio, Arttu Peuna, Riikka Korja, Mikko Aro, Paavo H.T. Leppänen, Jetro J. Tuulari, Harri Merisaari

**Affiliations:** a FinnBrain Birth Cohort Study, Turku Brain and Mind Center, Department of Clinical Medicine, University of Turku, Turku, Finland; b Centre for Population Health Research, Turku University Hospital and University of Turku, Turku, Finland; c Centre of Excellence in Learning Dynamics and Intervention Research (InterLearn), University of Jyväskylä and University of Turku, Finland; d Department of Psychology and Education, University of Jyväskylä, Finland; e Department of Clinical Medicine, Unit of Public Health, University of Turku, Finland; f Department of Child Psychiatry, Turku University Hospital, Turku, Finland; g Department of Radiology, Turku University Hospital and University of Turku, Turku, Finland; h Department of Medical Physics, Turku University Hospital and University of Turku, Turku, Finland; i Department of Diagnostic Services, Hospital Nova of Central Finland, Wellbeing Services County of Central Finland, Finland; j Department of Psychology and Speech-Pathology, University of Turku, Finland; k Department of Education, University of Jyväskylä, Finland; l Turku Collegium for Science and Medicine, University of Turku, Turku, Finland; m Department of Psychiatry, Turku University Hospital and University of Turku, Turku, Finland

**Keywords:** FinnBrain Protocol, Neuroimaging, MRI, Multi-centre studies, Reproducibility, Multi-scanner, Multi-vendor

## Abstract

Large multi-site studies that combine magnetic resonance imaging (MRI) data across research sites present exceptional opportunities to advance neuroscience research. However, scanner or site variability and non-standardised image acquisition protocols, data processing and analysis pipelines can adversely affect the reliability and repeatability of MRI derived brain measures. We implemented a standardised MRI protocol based on that used in the Adolescent Brain Cognition Development (ABCD)^Ⓡ^ study in two sites, and across four MRI scanners.

Twice repeated measurements of a single healthy volunteer were obtained in two sites and in four 3T MRI scanners (vendors: Siemens, Philips, and GE). Imaging data included anatomical scans (T1 weighted, T2 weighted), diffusion weighted imaging (DWI) and resting state functional MRI (rs-fMRI). Standardised containerized pipelines were utilised to pre-process the data and different image quality metrics and test-retest variability of different brain metrics were evaluated.

The implementation of the MRI protocols was possible with minor adjustments in acquisition (e.g. repetition time (TR), higher b-values) and exporting (DICOM formats) of images due to different technical performance of the scanners. This study provides practical insights into the implementation of standardised sequences and data processing for multisite studies, showcase the benefits of containerised preprocessing tools, and highlights the need for careful optimisation of multisite image acquisition.

## Graphical abstract

**Figure:**
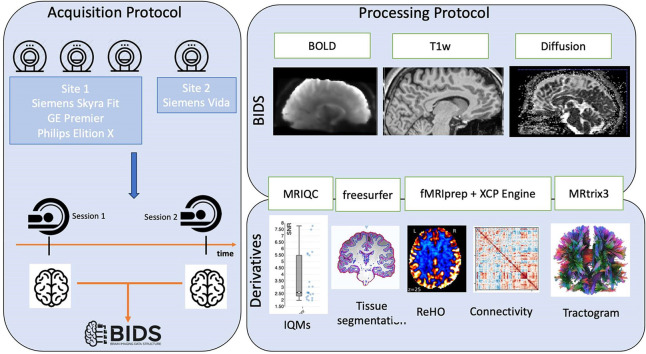

Specifications table

**Table:** 

Subject area:	Neuroscience
More specific subject area:	Neuroimaging
Name of your protocol:	FinnBrain protocol
Reagents/tools:	N-A
Experimental design:	Pilot study
Trial registration:	If applicable, include clinical trial registry and number
Ethics:	Ethical permission was obtained from the Ethics Committee of the Hospital District of Southwest Finland (VARHA/18,203/13.02.02/2023) and written informed consent was obtained from the subject prior to the experiment.
Value of the Protocol:	• The protocol provides practical insights into the implementation of standardised sequences and data processing for multisite studies. • This showcases the benefits of containerised preprocessing tools and highlights the need for careful optimisation of multisite image acquisition.

## Background

Large-scale longitudinal and multi-site studies are important for almost all areas of brain research including exploration neuroimaging markers of normal ageing, neurodegenerative and mental health disorders [[1], [2], [3]]. Longitudinal studies can generate important insights into disease progression and outcomes that are not available in cross-sectional studies, while multi-centre studies make larger sample sizes possible. Pertaining to neuroimaging, various large multi-centre data exist, e.g. the Adolescent Brain Cognition Development (ABCD) study [4], UK Biobank [5], Alzheimer's Disease Neuroimaging Initiative (ADNI) [6] etc. These studies have contributed significantly to promoting collaborations across institutions and countries, and experts worldwide can access these large data sets as evident, for example, human brain growth study [7]. In Finland, the ongoing Finnbrain [8] Birth Cohort studies the effects of prenatal and early life experiences (e.g. exposure to stress) on child brain development and health (*N* = 3808 families). This work is motivated by the next wave of data collection that will take place at age 10–11 years, and the inclusion of additional data collections as part of The Centre of Excellence for Learning Dynamics and Intervention Research (InterLearn CoE: https://interlearn.fi/en).

There are multiple important challenges associated with the acquisition of MRI data in large-scale multi-site studies. Previous reproducibility studies of structural MRI have demonstrated that changes in variables, such as scan session or MRI sequence [9] and head motion during scanning [10] may adversely affect brain measurement reliability. Likewise, pre-processing pipelines have an effect on segmentation [11] and morphometric measurements such as cortical thickness [12]. Additionally, multiple studies demonstrated significant variability in diffusion tensor imaging (DTI) metrics, such as fractional anisotropy (FA) and mean diffusivity (MD), across different scanners [[13], [14], [15]]. The reliability of functional MRI is affected not only by the acquisition and pre-processing strategies [16] but also due to variability across brain networks that are captured during the measurement [17] and differences between populations [18]. In addition, inter-vendor and test-retest reproducibility of resting-state functional magnetic resonance imaging (rs-fMRI) [19] along with longitudinal reproducibility of network connectivity [20] have also been investigated. Results from these studies implies that quality assurance metrics of raw images (for example signal- and contrast-to-noise ratio, and repeatability) and image analysis results must be rigorously compared across MRI vendors and sites.

Here, we implemented a standardised ABCD^Ⓡ^ MRI protocol on four different MRI scanners and evaluated the reproducibility and reliability of structural, rs-fMRI and DTI measurements. This yields insights to the impact of scanner differences on commonly used MRI metrics and identify potential sources of variability that can affect the interpretation of MRI data.

## Description of protocol

### Image acquisition

**Fig. 1:**
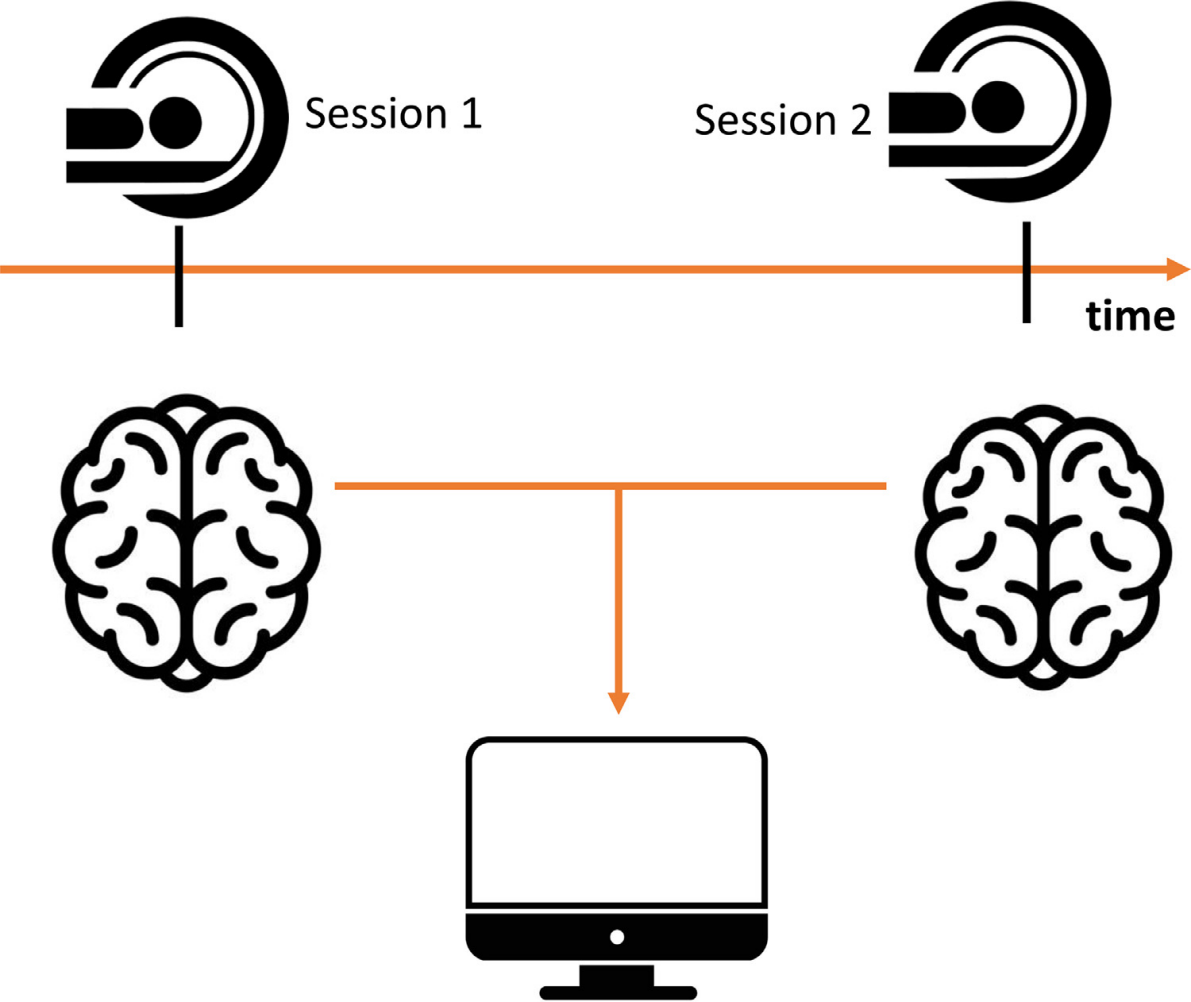
Depiction of the study design: Single subject was scanned twice in one day on three 3T scanners (Siemens MAGNETOM Skyra fit, GE Signa Premier, Philips Ingenia Elition X) at site 1 and one scanner (Siemens MAGNETOM Vida) at site 2. Data from both sessions was then pre-processed and analysed to evaluate reliability and test-retest repeatability of different image derived metrics.

One healthy volunteer (Male, Age = 27 years) was scanned twice using four 3T MRI scanners as follows: site 1 - Turku University Hospital (TYKS), Turku; Signa Premier (GE Healthcare, Milwaukee, WI), MAGNETOM Skyra fit (Siemens Healthcare, Erlangen, Germany) and Ingenia Elition X (Philips Healthcare, Best, Netherlands) and site 2 - Hospital Nova of Central Finland, Jyväskylä; MAGNETOM Vida (Siemens Healthcare, Erlangen, Germany). Two sessions for each scanner were performed on the same day with a 15-minute interval between the two sessions (Fig. 1). Multi-channel receiver coils were used in the experiments as follows: 32 channels (head only) in the Philips scanner and 48 and 64 channels (head and neck) in the GE and Siemens scanners, respectively. During the resting-state acquisitions, no specific cognitive tasks were performed, and the volunteer was instructed to keep eyes closed, minimise eye movements, and relax inside the scanner.

Imaging protocol included structural MRI series (T1w and T2w images), one diffusion MRI series (with *b* = 0, 500 (6 directions), 1000 (15 directions), 2000 (15 directions), 3000 (60 directions); total directions = 96) and one rs-fMRI. Imaging parameters were made as similar as possible across scanner manufacturers, although some hardware and software constraints were unavoidable. Details of image acquisition parameters of imaging protocol are described in prior work of the ABCD study group [21]. Each scan session took approximately 40 min to complete and was tolerated well by the volunteer.

## Challenges in the implementation of ABCD study protocol

Following were the challenges faced while implementing ABCD study protocol in different scanners:

1. **Logistics:** Finding a volunteer to scan eight times including a visit to another city and coordination. However, all the scans were acquired within a span of two weeks.
2. **Setting the same acquisition parameters across all scanners:** For MAGNETOM Skyra fit, minimum repetition time (TR) that could be achieved was around 2000 ms resulting in long acquisition time and less blood-oxygen-level-dependent (BOLD) volumes (200) as compared to other scanners (380). We received the ABCD compatible sequences for Siemens from another ABCD study site that had better gradients compared to MAGNETOM Skyra fit). Hence, the sequence was incompatible with our scanner leading to longer TR in EPI sequences (see point 3).
3. **Higher b-value diffusion MRI:** For MAGNETOM Skyra fit with *b* = 3000, TR could not be decreased resulting in very long acquisition time for this sequence (30 min).
4. **Inconsistencies with DICOM export and conversion:** We noted some inconsistencies in DICOM export for GE Signa that subsequently affected the Nifti conversion. This is not ideal for collecting large datasets and can result in data loss. In addition, Siemens Vida enhanced DICOM format was challenging to convert into brain imaging data structure BIDS standard with commonly used tools.

### Scanner data post-processing

After each scan, all the images were sent to local Picture Archiving and Communication System (PACS) and then transferred to local database system for further processing. Data were converted to standardised Brain Imaging Data Structure (BIDS) [22] before further processing. All scans were visually checked before starting the analyses. All the processing was performed on one computer with the same set of software.

### Image quality metrics (IQMs)

Image quality metrics (IQMs) were derived to assess the quality of structural and functional MRI with MRIQC [23] (https://mriqc.readthedocs.io/en/latest/measures.html). MRIQC is an auto-processing pipeline that can easily be used and provide quantifiable image quality metrics. For both structural and rs-fMRI, a subset of IQMs were included: contrast to noise ratio (CNR), full width at half maximum (FWHM) and overall signal to noise ratio (SNR). For rs-fMRI, temporal signal-to-noise ratio (tSNR) to measure quality of BOLD signal; framewise displacement (FD) to measure head motion; FWHM as a measure for spatial information and differential variation in the signal (DVARS) as a measure of temporal signal variation was used. These IQMs were compared between different scanners as well as between session (will be referred as session 1 = test, session 2 = retest) of each scanner to assess test-retest repeatability of IQMs.

### Structural MRI pre-processing

The T1w test-retest scans were processed using FreeSurfer version 6.0 for automatic brain segmentation and parcellation [24]. FreeSurfer basic pre-processing included affine registration of raw T1w images 1 mm [3] template (MNI305), and after normalisation, removal of intensity bias-field and skull stripping, white matter voxels are identified based on intensity and neighbour constraints. Cortical thickness at each vertex was computed as the average of two distances, that is, from each vertex in the grey-white surface to the nearest point in pial surface and from the corresponding vertex in the pial surface to the nearest point in grey–white surface [25]. Global and regional brain measures of subcortical volume, cortical volume, cortical thickness, and cortical surface area were extracted and were compared between test-retest scans for each scanner. Variability between brain volume and cortical thickness between test-retest scans was calculated as: VAR = 100*((test-retest)/avg(test, retest)). We processed the surfaces to same space with MNE-python package (version 1.4.0) [26] for repeatability analysis.

### Resting state functional MRI (rs-fMRI) pre-processing

Pre-processing of rs-fMRI data was carried out using fMRIPrep [27] version 20.2.1. The following steps were applied to all datasets: skull stripping, motion correction, slice time correction, susceptibility distortion correction, and co-registration of the functional and anatomical scans. As field maps or reverse phase encoding acquisitions were not available, we adopted a field map-less susceptibility for distortion correction procedure which is based on nonlinear registration of the EPI images to the same-subject T1w images [28]. Independent component analysis - Automatic removal of motion artifacts (ICA-AROMA) [29] was used to denoise BOLD time-series. The output of fMRIPrep was specified to be in MNI152NLin2009Asym template for further processing.

The pre-processed fMRIPrep output was then further processed with eXtensible Connectivity Pipeline Engine (xcpEngine) [30]. xcpEngine also employs basic preprocessing of structural and functional data followed by denoising of fMRI signal and then estimates voxel-wise regional homogeneity (ReHo), amplitude of low-frequency fluctuations (ALFF) and functional connectivity between each pair of regions in several brain atlases. ReHo and ALFF values from Automated anatomical labelling (AAL) atlas were compared between sessions for each scanner. We used the standard 36P denoising approach that entails frame-to-frame motion estimates, mean signals from white matter and cerebrospinal fluid, the mean global signal, and quadratic and derivative expansions of these signals [31]. To evaluate a bias between the mean difference and to estimate an agreement interval in ReHo and ALFF maps, the Bland-Altman plot was used [32]. In addition, functional connectome matrices from each session of scanner were also compared.

Group level ICA was carried out for the xcpEngine denoised fMRI data using Probabilistic ICA [33] as implemented in MELODIC (Multivariate Exploratory Linear Decomposition into Independent Components) version 3.15, part of FSL (FMRIB's Software Library, www.fmrib.ox.ac.uk/fsl). For this analysis, Siemens Skyra fMRI was not included as the number of BOLD volumes and TR was not same to the rest of the scanners as mentioned above. Because of the difference in the TR, analysis could not be performed after discarding the volumes from other three datasets. The following data pre-processing was applied to the input data: masking of non-brain voxels; voxel-wise de-meaning of the data; normalisation of the voxel-wise variance. Pre-processed data were whitened and projected into a 63-dimensional subspace using probabilistic Principal Component Analysis (PCA) where the number of dimensions was estimated using the Laplace approximation to the Bayesian evidence of the model order [34]. Estimated component maps were divided by the standard deviation of the residual noise and thresholded by fitting a mixture model to the histogram of intensity values [33]. FSL's dual regression followed by paired *t*-test was performed to see the difference between brain networks across scanner and between test-retest scans.

### Diffusion MRI pre-processing

FSL (version 6.0) in combination with MRtrix [35] (version 3.0.2) was used to pre-process the DWI data. Pre-processing included dMRI noise level estimation and denoising using Marchenko-Pastur PCA [36] followed by correction for Gibbs ringing using the method of local subvoxel-shifts [37]. Diffusion image pre-processing was performed including eddy current-induced distortion correction, motion correction, and susceptibility-induced distortion correction, using FSL's eddy [38], topup [39] and applytopup tools.

Tract-Based Spatial Statistics (TBSS) were used with the default settings to create a skeletonised version of the FA and MD values (computed using FSL's DTIFit) that were fitted to the pre-processed multi-shell diffusion data. Global FA and MD values were computed for all skeleton voxels. In addition, average FA and MD values were computed over skeleton voxels from 20 regions of interest (ROIs) selected from the JHU white matter tractography atlas [40]. Variability between FA values of test and re-test scans was calculated as VAR= 100*((test-retest)/avg(test, re-test). We transformed the FA scalar maps to MNI space using ants_tbss package (https://github.com/trislett/ants_tbss) for visualisation of variability values with our in-house software.

## Protocol validation

### Structural MRI

**Fig. 2:**
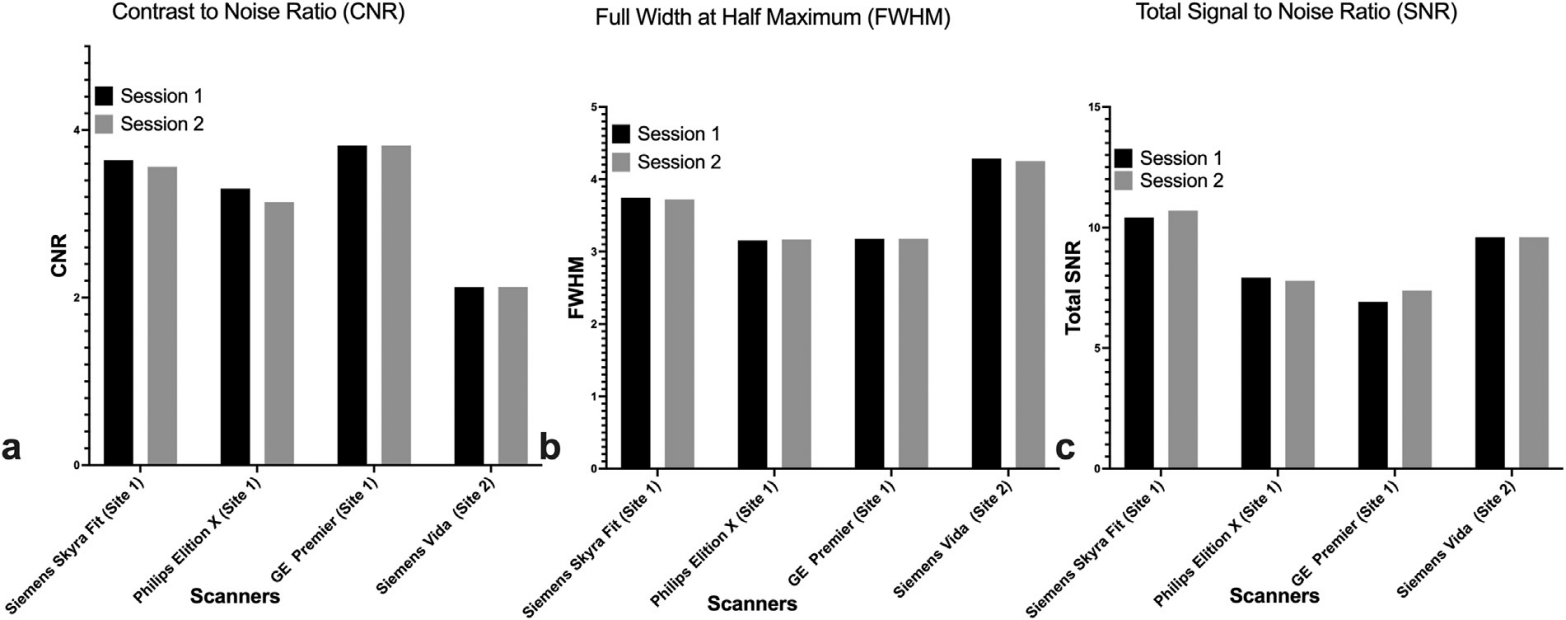
Bar graph showing image quality metrics IQMS a) Contrast to Noise Ratio (CNR), b) Full Width at Half Maximum (FWHM) and c) Total Signal to Noise Ratio (SNR) calculated using MRIQC pipeline for all four scanners and two sessions.

The comparison of the IQMs derived from structural MRI between different scanners and between two sessions of each scanner is shown in Fig. 2. Between different scanners, there was some variability amongst all three IQMs. The CNR for all scanners (test; re-test): Siemens Skyra Fit (3.64; 3.56), Philips Elition X (3.3; 3.13), GE Premier (3.81, 3.82), Siemens Vida (2.12; 2.12).

• The FWHM for all scanners (test; re-test): Siemens Skyra Fit (3.74; 3.72), Philips Elition X (3.15; 3.16), GE Premier (3.15; 3.17), Siemens Vida (4.28; 4.24).
• The overall SNR was variable across all scanners (test; re-test): Siemens Skyra Fit (10.4; 10.69), Philips Elition (7.91; 7.79), GE Signa (6.91; 7.39), Siemens Vida (9.59; 9.60).

Overall, CNR, FWHM and SNR between sessions were not different and showed good test-re-test repeatability.

**Fig. 3:**
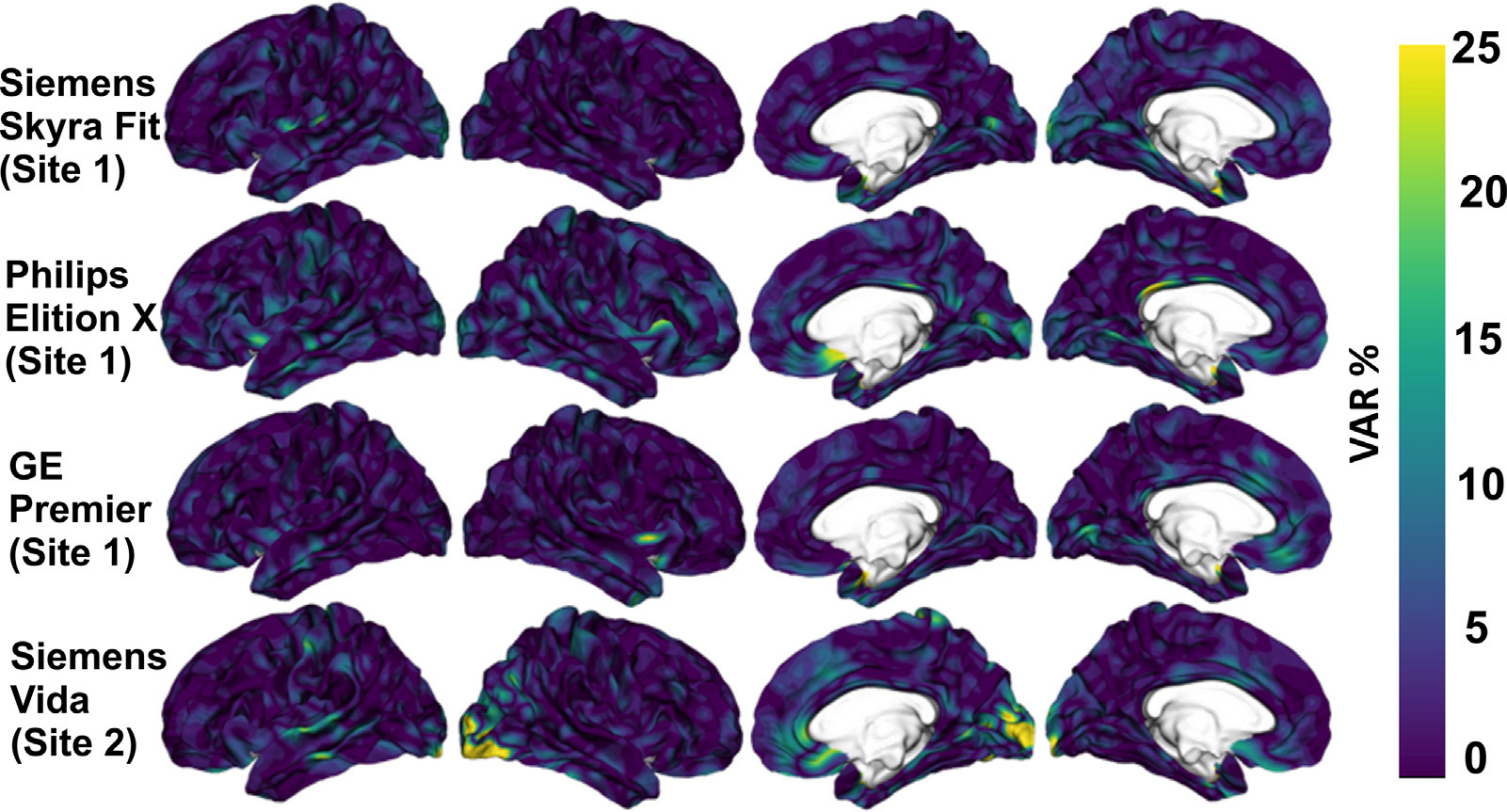
Variability VAR= 100*((test-retest)/avg(test, re-test)) of cortical thickness values with 10 mm FWHM smoothing.

Fig. 3 shows variability values for the four evaluated scanners, when the thickness estimates were smoothed with 10 mm FWHM filter. Apart from small region in occipital lobe in Siemens Vida data (15–25 %), the thickness estimates were considered good (VAR < 10 %).

Variability between cortical thickness and brain volumes from different ROIs using FreeSurfer is shown in Supplementary Fig. S1. For cortical thickness, the entorhinal region showed the highest variability greater than 15 % whereas rest of the regions showed variability of less than 10 % (Fig. S1a). For main volumes, the test-retest variability amongst two sessions was highest for cerebral white matter and subcortical grey matter whereas rest of the volumes showed variability of less than 5 % (Fig. S1b). Variability of white matter volume was the highest in our study and can be attributed to the fact that areas of brain with higher myelin content also show higher variability due to shifting of the apparent grey–white boundary [12]. Difference in the reproducibility of FreeSurfer segmentation is known and is affected by various factors such as type of sequence, number of receiver coil channels [41] and also version of FreeSurfer [42].

### Functional MRI

**Fig. 4:**
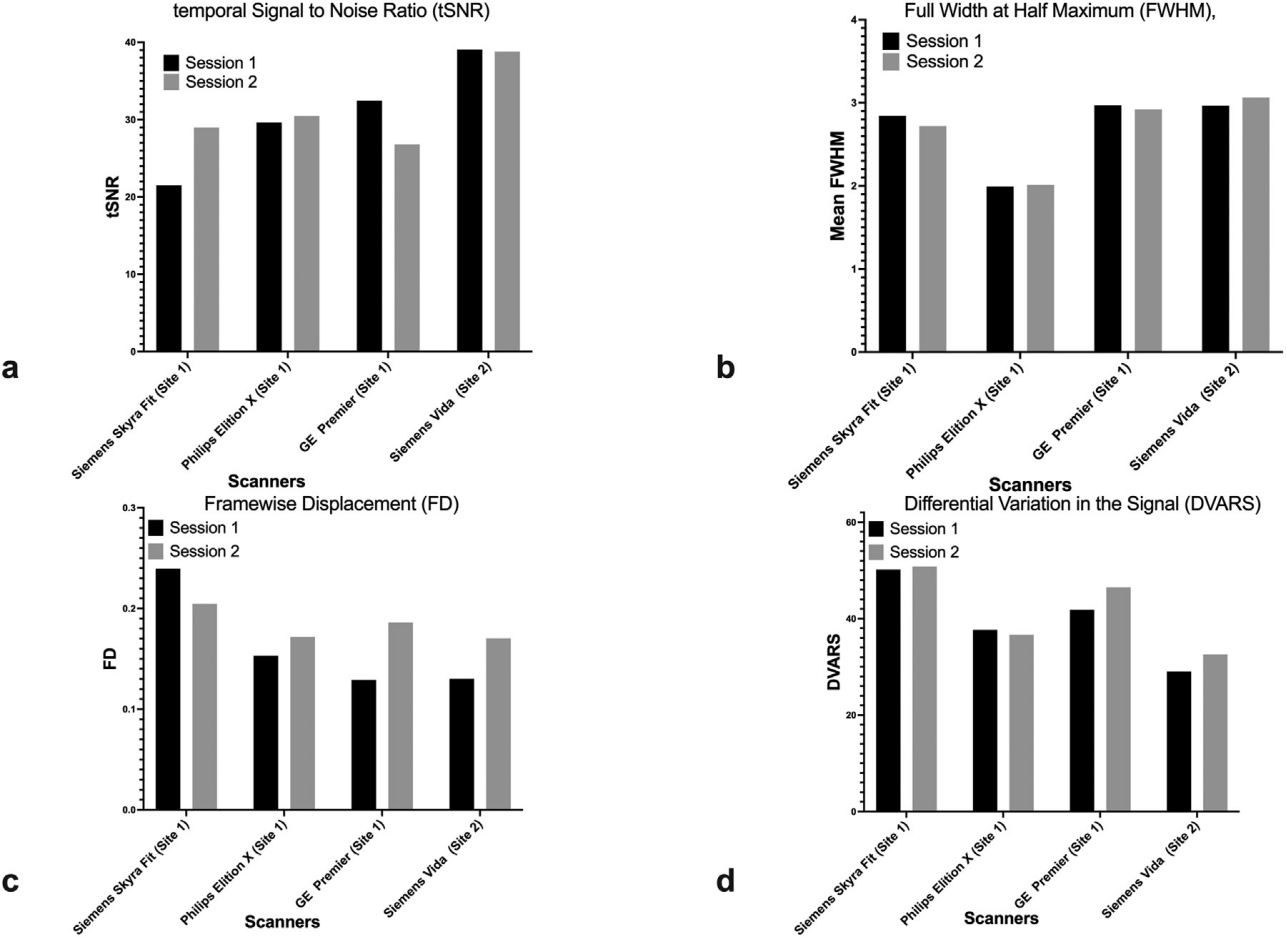
Bar graph showing image quality metrics IQMS a) temporal Signal to Noise ratio (tSNR), b) Full Width at Half Maximum (FWHM), c) Framewise Displacement (FD) and d) differential variation in the signal (DVARS) for all four scanners and two sessions.

There were considerable differences in IQMs derived from MRIQC across all scanners and between sessions (Fig. 4).

• The tSNR for test and re-test had more difference for Siemens Skyra Fit (21.49; 28.96) and GE Premier (32.46; 26.78) as compared to Philips Elition X (29.63; 30.47) and Siemens Vida (39.06; 38.80). Across sites, tSNR is expected to be affected with variable number of time points in the series [43], whereas within site the variability can be due to in thermal and physiological noise sources [44].
• For FWHM, the test-retest values were close to each other. However, differences across scanners were evident: Siemens Skyra Fit (2.84; 2.71), Philips Elition X (1.99; 2.01), GE Premier (2.97; 2.92), Siemens Vida (2.96; 3.06). A major cause of site differences in FWHM could be due to the use spatial filtering during the image reconstruction process in k-space. Previous studies investigating inter-vendor differences demonstrated that images from some vendors were smoother than the others owing to difference in k-space filtering algorithm [45]. This can be addressed by using harmonisation pipeline or by using filter in the postprocessing to equalise the blurring.
• DVARS for all scanners was as follows: Siemens Skyra Fit (50.18; 50.80), Philips Elition X (37.69; 36.67), GE Premier (41.82; 46.51), Siemens Vida (29.04; 32.57). Since DVARS is associated with temporal signal variations beyond those reflected in head motion it is more likely to be affected by physiological noise such as cardiac pulsation and respiratory rate variability [46].
• FD for all scanners showed good test-retest repeatability: Siemens Skyra Fit (0.23; 0.20), Philips Elition X (0.15; 0.17), GE Premier (0.12; 0.18), Siemens Vida (0.13; 0.17). Variability in the FD across site can be attributed to the type and the number channels in the head coil used.
• Notably, FD and DVARs also varied amongst scanners and between two sessions, which explains some of the variability in IQM's. The inter-site differences in IQMs related to subject motion (DVARS and FD) are relatively complex and makes challenging to interpret the findings. However, challenges pertaining to these motion related confounding factors can be addressed in the preprocessing by employing relevant motion correction or by using the FD as a regressor in analysis to mitigate residual motion-related effects [47]. It should be noted that certain inevitable differences owing to vendors such as channel number of receiver coils, differences in the gradient system, and magnetic field inhomogeneity can have an influence on the quality of data.

**Fig. 5:**
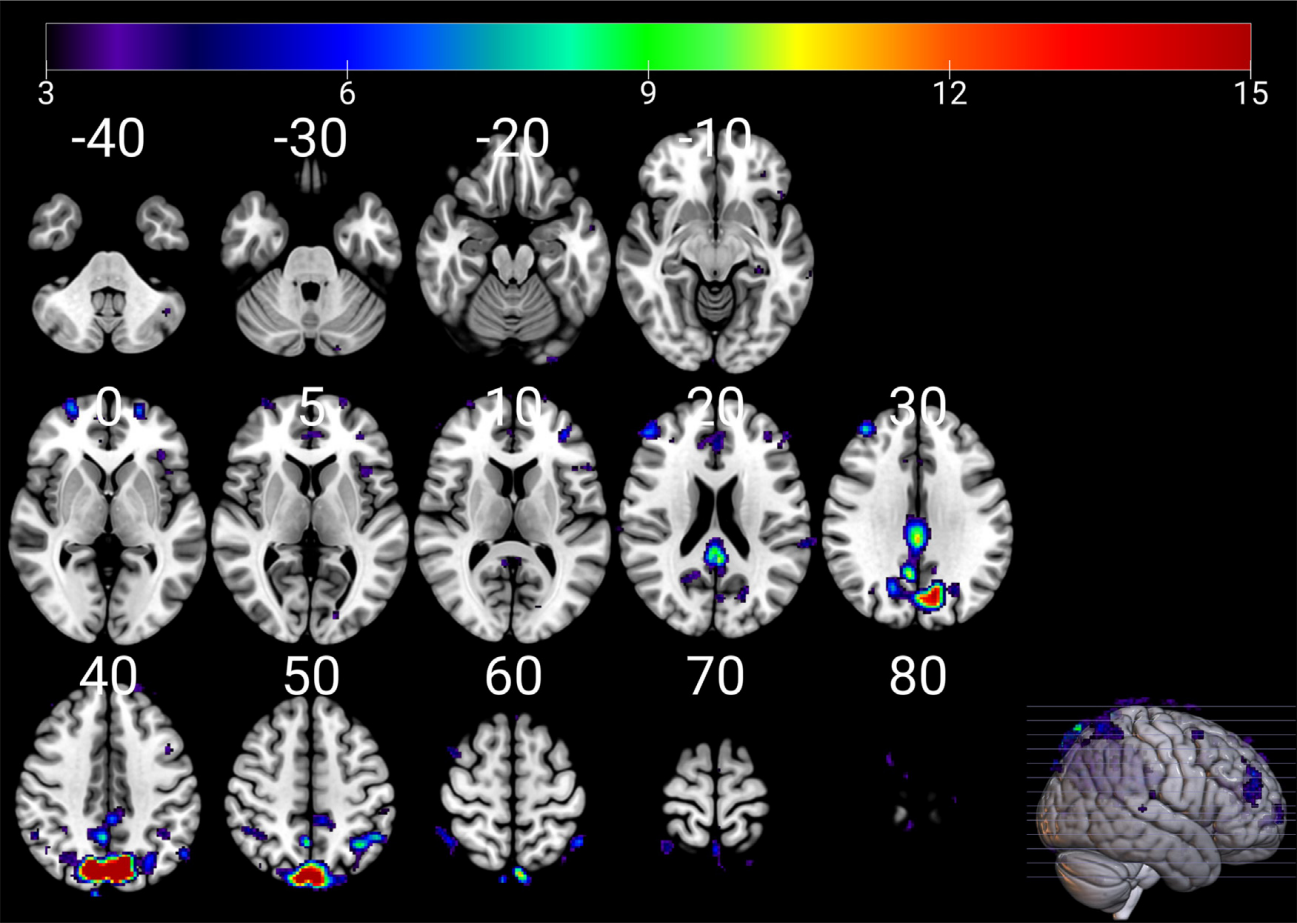
‘Group ICA’ across all scans showing the activation of areas corresponding to default mode network (DMN). The figure was generated with mriCroGL.

Fig. 5 shows the common brain networks from three scanners. Dual regression did not show any significant differences between nodes across all scanners and sessions (Supplementary Fig. 2). A study investigating the inter-vendor and test-retest reliabilities of resting-state functional magnetic resonance imaging also demonstrated resting state fMRI as a reliable imaging marker [47]. ICA is one of the most commonly used methods for analysis of resting state fMRI and has shown moderate to good test-retest reliability for intrinsic connectivity networks [48]. Although the current study used the same subject for all the scanners, the possibility of innate variability in the resting state brain network of the subject from one scan to another cannot be excluded.

Variability between ReHo (Supplementary Fig. 3) and ALFF (Supplementary Fig. 4) values of two sessions and four scanners were evaluated using Bland-Altman. Results of the Bland-Altman plot showed ReHo values for Siemens Skyra Fit were lowest because of less BOLD volumes as compared to rest of the scanners.

• ReHo mean difference calculated with each scanner's fMRI were 0.0039, 0.0005, −0.0007 and −0.01 for Siemens Skyra Fit, Philips Elition X, GE Premier, and Siemens Vida, respectively. The variability of ReHo values as a function of functional time points has also been reported in previous studies [49] and explains why ReHo values in present study for Siemens Skyra (time points = 200) was less as compared to other scanners (time points = 380).
• ALFF values showed significantly larger variations amongst all the scanners with ALFF mean of 476, 210,304, 1480, 41.76 for Siemens Skyra Fit, Philips Elition X, GE Premier and Siemens Vida respectively. Large variability in the ALFF values across scanners is consistent with the previous study investigating the inter-site reliability of voxel wise brain analytics measure such as ReHo and ALFF [50]. The difference in the variable mean ALFF values across scanners can be attributed to the unequal scaling of the BOLD signal employed by different vendors difference and can be reduced by rescaling the signal intensity [51].

### Diffusion MRI

**Fig. 6:**
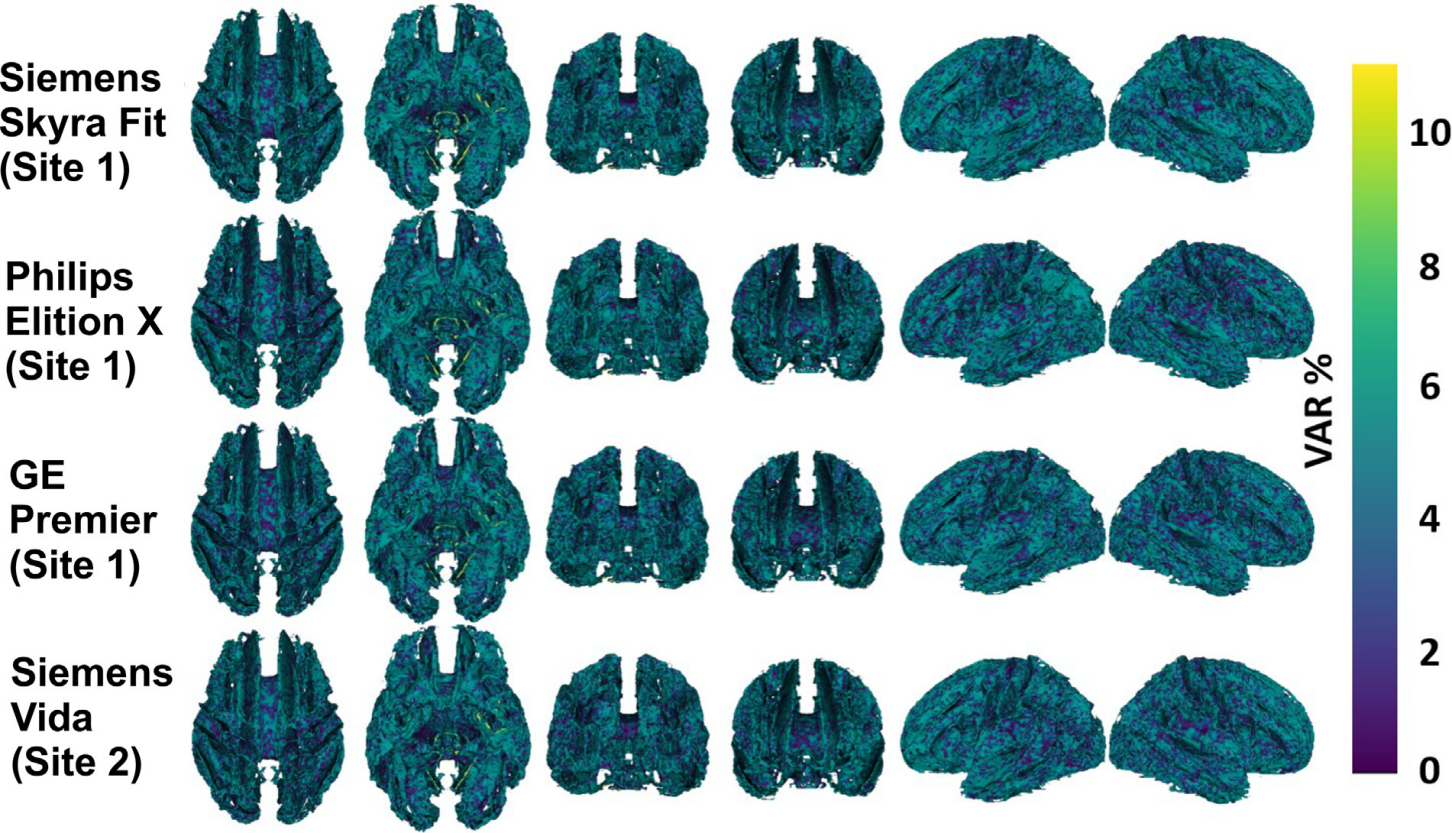
Variability VAR= 100*((test-re-test)/avg(test, re-test)) of Diffusion Tensor Imaging Fractional Anisotropy values in four scanner models. The shown values were calculated for each voxel in Tract Based Statistical Analysis skeleton, which is also the underlay in the figure.

Fig. 6 shows variability values for the four evaluated scanners. All the FA values were highly repeatable (VAR < 10 %) in all the data.

ROI-wise variability between FA values of two sessions from each scanner are shown in Supplementary Fig. 5. For diffusion derived metrics like FA, the current study established that inter-scanner variability is non-uniform across the white matter of the human brain, with a variability up to 5 %. Similar range of variability in diffusion metrics of major brain tracts has also been reported earlier as well [[52], [53]]. Recently, investigators have examined the reproducibility of multi-shell diffusion images in a multi-site study involving travelling subject [15]. A 7.7 % median inter-center coefficient of variation was estimated for the track density maps in whole white matter among the subjects. Fundamentally, multi-site reproducibility is likely to suffer from inaccuracy and biases either from variety of MRI hardware or from inconsistent acquisition protocols [54]. Previous studies have shown that the SNR [55], Echo Time (TE) and b-values [56] tend to affect the diffusion derived measurements. While measurements with higher b-values (e.g., 2000 s/mm^2^ or even 3000 s/mm^2^) would be more useful for differentiating complex white matter, they could also be a substantial challenge due to gradient hardware limit [57].

In recent years, there has been growing concern about the reproducibility of neuroimaging data, and several studies have been conducted to investigate this issue. These studies have highlighted the variability in MRI data across different imaging sites and scanner vendors and have identified factors that contribute to this variability. When comparing values across different sites, variability in imaging metrics remains relatively high warranting the need to develop more reliable, sensitive, and specific measurements. In this realm, the current study presents a protocol for implementing ABCD MRI protocol in different scanners and evaluated the quality of data with standardised easy-to-implement processing pipelines. Based on our experience of implementing ABCD sequence across multi-sites and different vendors, our recommendations are as follows:

1. The study design, specifically the use of a single patient, makes generalisability of the current study more challenging. To obtain statistically meaningful measures regarding the reproducibility, a bigger sample size should be used. However, organising travel visits with more subjects can be a logistics challenge in case the sites are situated at long distances.
2. Same vendors can have sequence compatibility issues due to the difference in the model of the scanner. Even subtle variation in image acquisitions parameters (as seen in Siemens Skyra Fit) can produce an unwanted source of variance that must be controlled.
3. Implementation of data QC and quality assurance in all the steps (image acquisition, data upload, pre-processing and post-processing) is important to ensure high-quality data.
4. The use of standardised and containerised pre-processing pipelines ensures that the similar processing steps are used. In addition, these established pipelines have plenty of support available from the experts and issues encountered can be addressed promptly.
5. Not only the sequences are very specific to scanner, the DICOM export is also unique to scanner type and model. E.g, new Siemens scanners all use enhanced data format (mosaic) as default. It is possible to configure them to use “interoperability” mode that simulates traditional DICOM. This will ensure that the DICOMs are easily converted into BIDS format.
6. Considerable attention must be paid for data organisation, processing, and availability to participating organisation and then openly as a resource so that the transparency of the multi-site data collection is ensured.

## Limitations

One of the limitations of the study is that there was only one subject and hence no statistical tests could be performed. However, the current scope of the study was to implement a neuroimaging protocol and evaluate the quality of the data without any inter-subject variability. Further studies with more subjects will be needed to support our findings. In addition, the subject was healthy young adult male whereas previous studies have shown that reproducibility of cortical thickness is affected by the age (i.e. lower reproducibility in children and adolescents than in adults) owing to non-linear brain maturation [58] and also by sex [59]. In addition, another fMRI study reported that old individuals showed low reliability of the resting-state functional connectivity [60]. Future studies with more heterogenous study subjects are warranted although inter-subject variability remains an issue in such investigations. Current study did not utilise any harmonisation approaches and future studies with methods such as ComBat [[54], [61]] can improve the inter-site variability.

## CRediT authorship contribution statement

**Wajiha Bano:** Conceptualization, Methodology, Software, Validation, Formal analysis, Data curation, Writing – original draft, Writing – review & editing, Visualization. **Elmo Pulli:** Conceptualization, Methodology, Software, Validation, Formal analysis, Investigation, Data curation, Writing – original draft, Writing – review & editing. **Lucia Cantonas:** Writing – review & editing. **Aino Sorsa:** Writing – review & editing. **Jarmo Hämäläinen:** Writing – review & editing, Project administration, Funding acquisition. **Hasse Karlsson:** Writing – review & editing, Project administration, Funding acquisition. **Linnea Karlsson:** Writing – review & editing, Project administration, Funding acquisition. **Ekaterina Saukko:** Investigation, Data curation, Writing – review & editing. **Teija Sainio:** Validation, Investigation, Data curation, Writing – review & editing. **Arttu Peuna:** Investigation, Data curation, Writing – review & editing. **Riikka Korja:** Writing – review & editing, Visualization, Funding acquisition. **Mikko Aro:** . **Paavo H.T. Leppänen:** Writing – review & editing, Project administration, Funding acquisition. **Jetro J. Tuulari:** Conceptualization, Methodology, Software, Validation, Investigation, Writing – review & editing, Visualization, Supervision, Project administration, Funding acquisition. **Harri Merisaari:** Conceptualization, Methodology, Software, Validation, Formal analysis, Investigation, Data curation, Writing – original draft, Writing – review & editing, Visualization, Supervision, Project administration, Funding acquisition.

## Declaration of competing interest

The authors declare that they have no known competing financial interests or Sconpersonal relationships that could have appeared to influence the work reported in this paper.

## Data Availability

Data will be made available on request.

## Appendix Supplementary materials

Image, application 1

Image, application 2

Image, application 3

Image, application 4

Image, application 5
