# Supplementary material for: Implementing ABCD studyⓇ MRI sequences for multi-site cohort studies: Practical guide to necessary steps, preprocessing methods, and challenges
Source: MethodsX. 2024 Jun 1;12:102789. doi: 10.1016/j.mex.2024.102789 (PMC11223117; doi:10.1016/j.mex.2024.102789)
Supplement: Supplementary file 1 [file mmc1.pptx]

## Slide 1
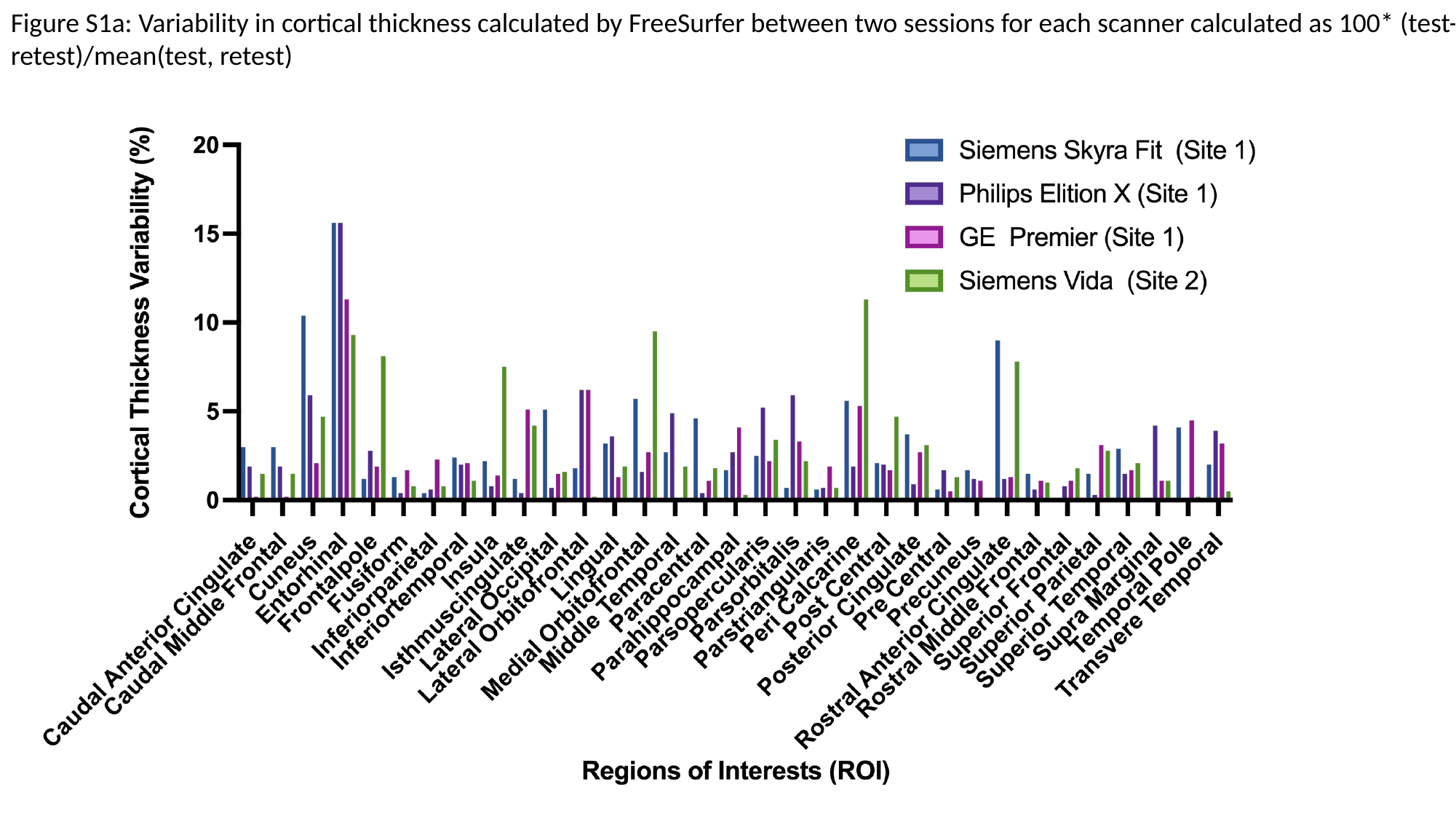

Figure S1a: Variability in cortical thickness calculated by FreeSurfer between two sessions for each scanner calculated as 100* (test-retest)/mean(test, retest)

## Slide 2
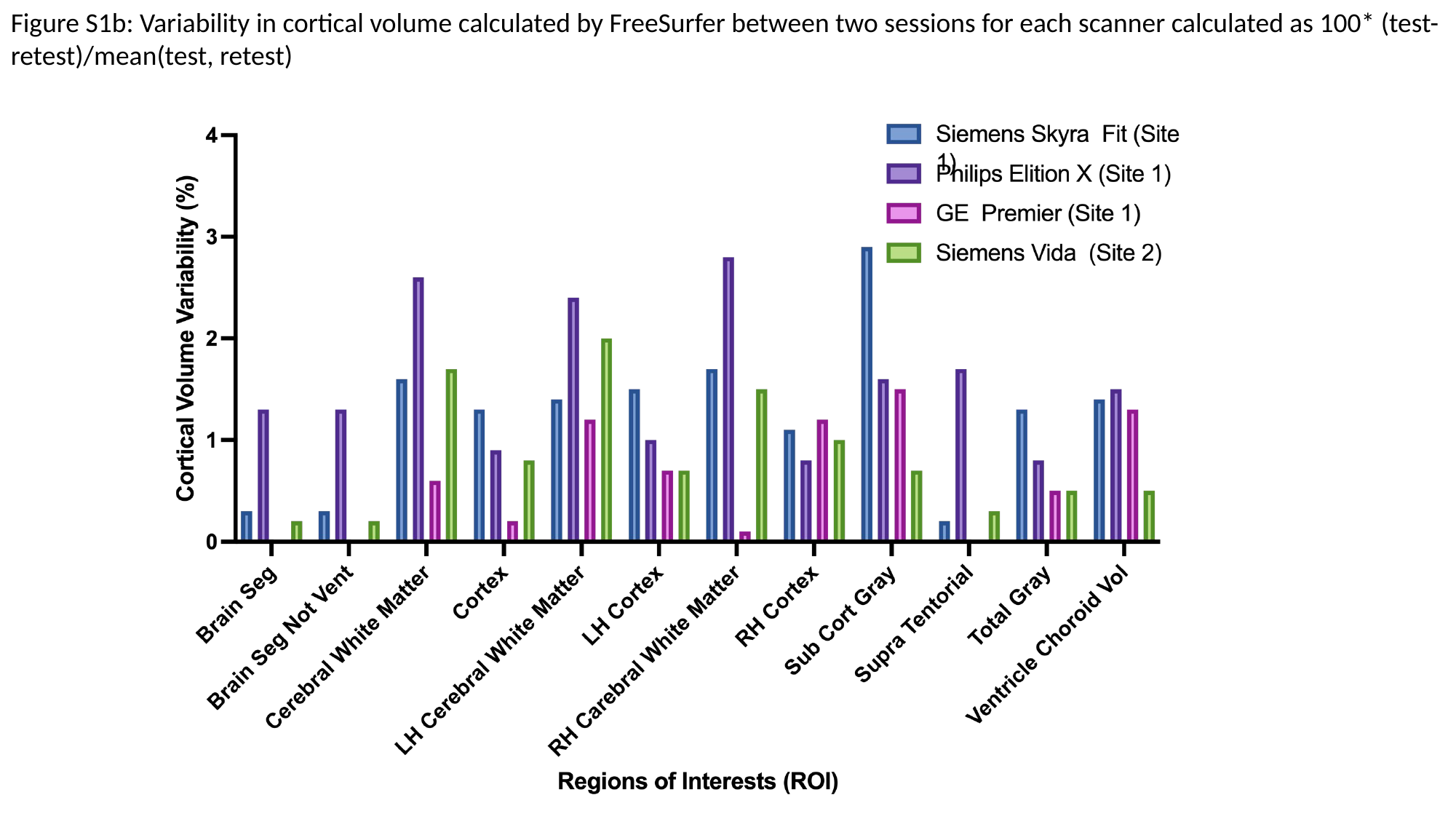

Figure S1b: Variability in cortical volume calculated by FreeSurfer between two sessions for each scanner calculated as 100* (test-retest)/mean(test, retest)
