# Supplementary material for: Implementing ABCD studyⓇ MRI sequences for multi-site cohort studies: Practical guide to necessary steps, preprocessing methods, and challenges
Source: MethodsX. 2024 Jun 1;12:102789. doi: 10.1016/j.mex.2024.102789 (PMC11223117; doi:10.1016/j.mex.2024.102789)
Supplement: Supplementary file 2 [file mmc2.pptx]

## Slide 1
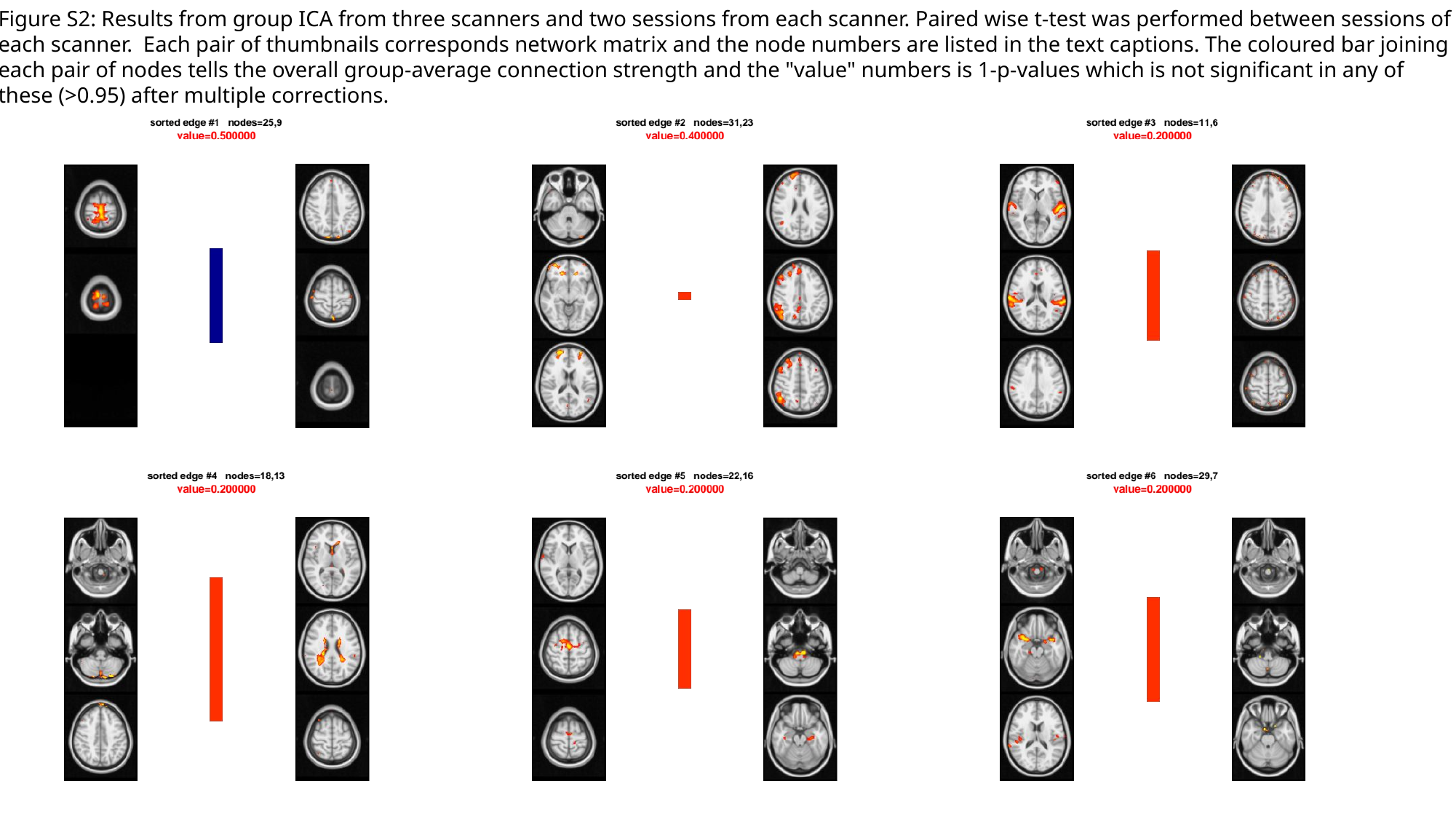

Figure S2: Results from group ICA from three scanners and two sessions from each scanner. Paired wise t-test was performed between sessions of each scanner. Each pair of thumbnails corresponds network matrix and the node numbers are listed in the text captions. The coloured bar joining each pair of nodes tells the overall group-average connection strength and the "value" numbers is 1-p-values which is not significant in any of these (>0.95) after multiple corrections.
