# Supplementary material for: Implementing ABCD studyⓇ MRI sequences for multi-site cohort studies: Practical guide to necessary steps, preprocessing methods, and challenges
Source: MethodsX. 2024 Jun 1;12:102789. doi: 10.1016/j.mex.2024.102789 (PMC11223117; doi:10.1016/j.mex.2024.102789)
Supplement: Supplementary file 3 [file mmc3.pptx]

## Slide 1
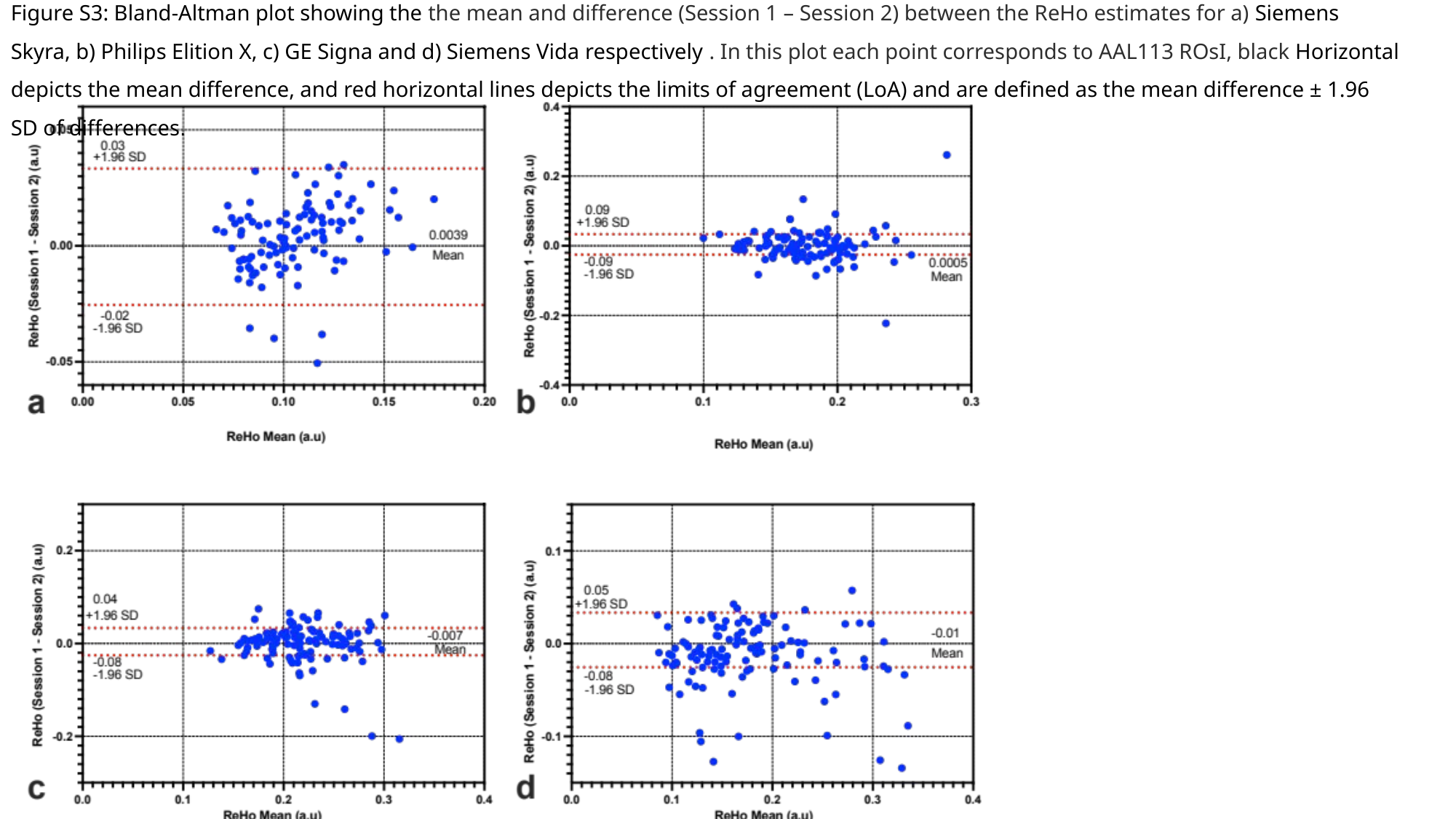

Figure S3: Bland-Altman plot showing the the mean and difference (Session 1 – Session 2) between the ReHo estimates for a) Siemens Skyra, b) Philips Elition X, c) GE Signa and d) Siemens Vida respectively . In this plot each point corresponds to AAL113 ROsI, black Horizontal depicts the mean difference, and red horizontal lines depicts the limits of agreement (LoA) and are defined as the mean difference ± 1.96 SD of differences.
