# Supplementary material for: Implementing ABCD studyⓇ MRI sequences for multi-site cohort studies: Practical guide to necessary steps, preprocessing methods, and challenges
Source: MethodsX. 2024 Jun 1;12:102789. doi: 10.1016/j.mex.2024.102789 (PMC11223117; doi:10.1016/j.mex.2024.102789)
Supplement: Supplementary file 4 [file mmc4.pptx]

## Slide 1
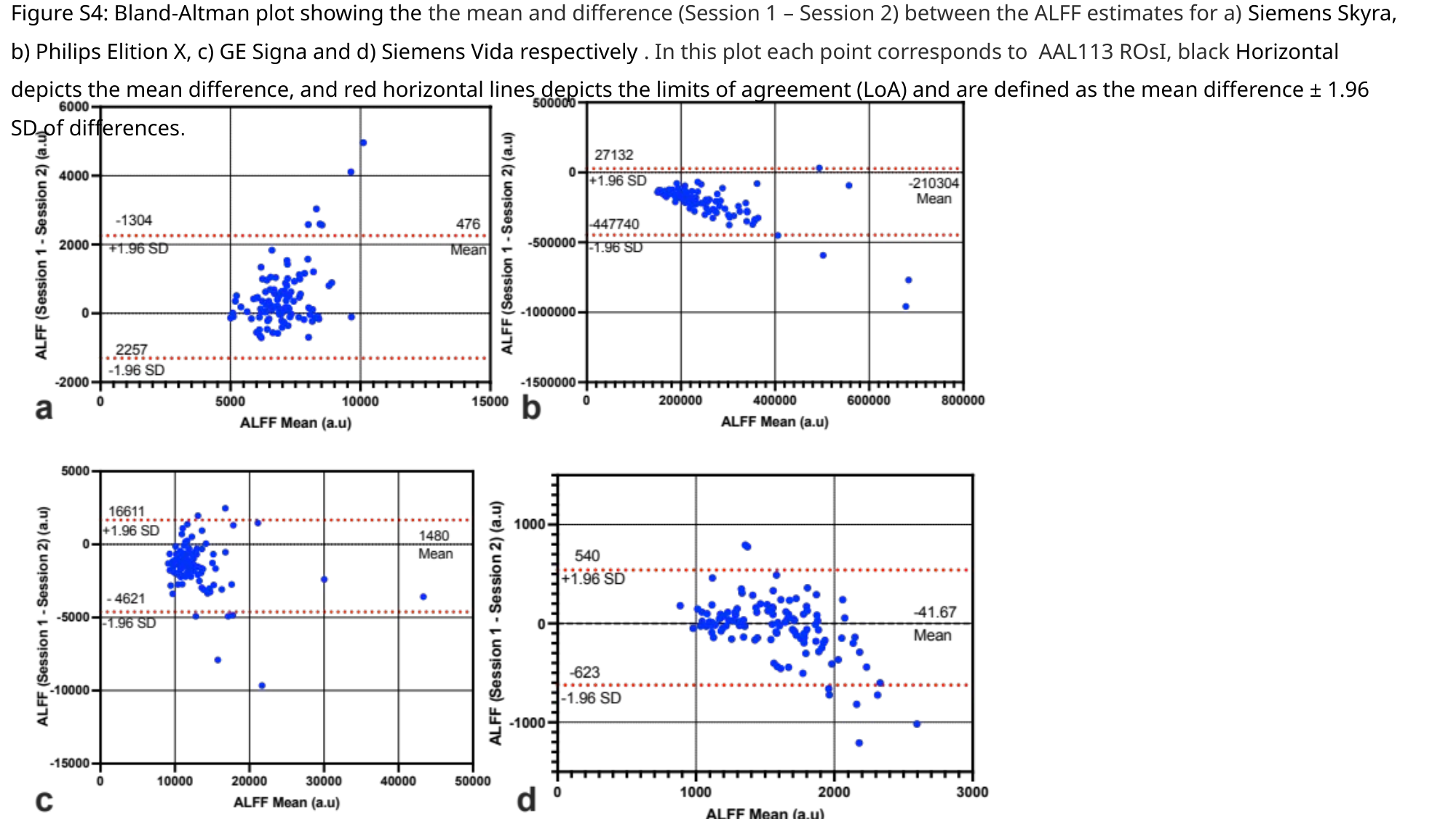

Figure S4: Bland-Altman plot showing the the mean and difference (Session 1 – Session 2) between the ALFF estimates for a) Siemens Skyra, b) Philips Elition X, c) GE Signa and d) Siemens Vida respectively . In this plot each point corresponds to AAL113 ROsI, black Horizontal depicts the mean difference, and red horizontal lines depicts the limits of agreement (LoA) and are defined as the mean difference ± 1.96 SD of differences.
