# Supplementary material for: Implementing ABCD studyⓇ MRI sequences for multi-site cohort studies: Practical guide to necessary steps, preprocessing methods, and challenges
Source: MethodsX. 2024 Jun 1;12:102789. doi: 10.1016/j.mex.2024.102789 (PMC11223117; doi:10.1016/j.mex.2024.102789)
Supplement: Supplementary file 5 [file mmc5.pptx]

## Slide 1
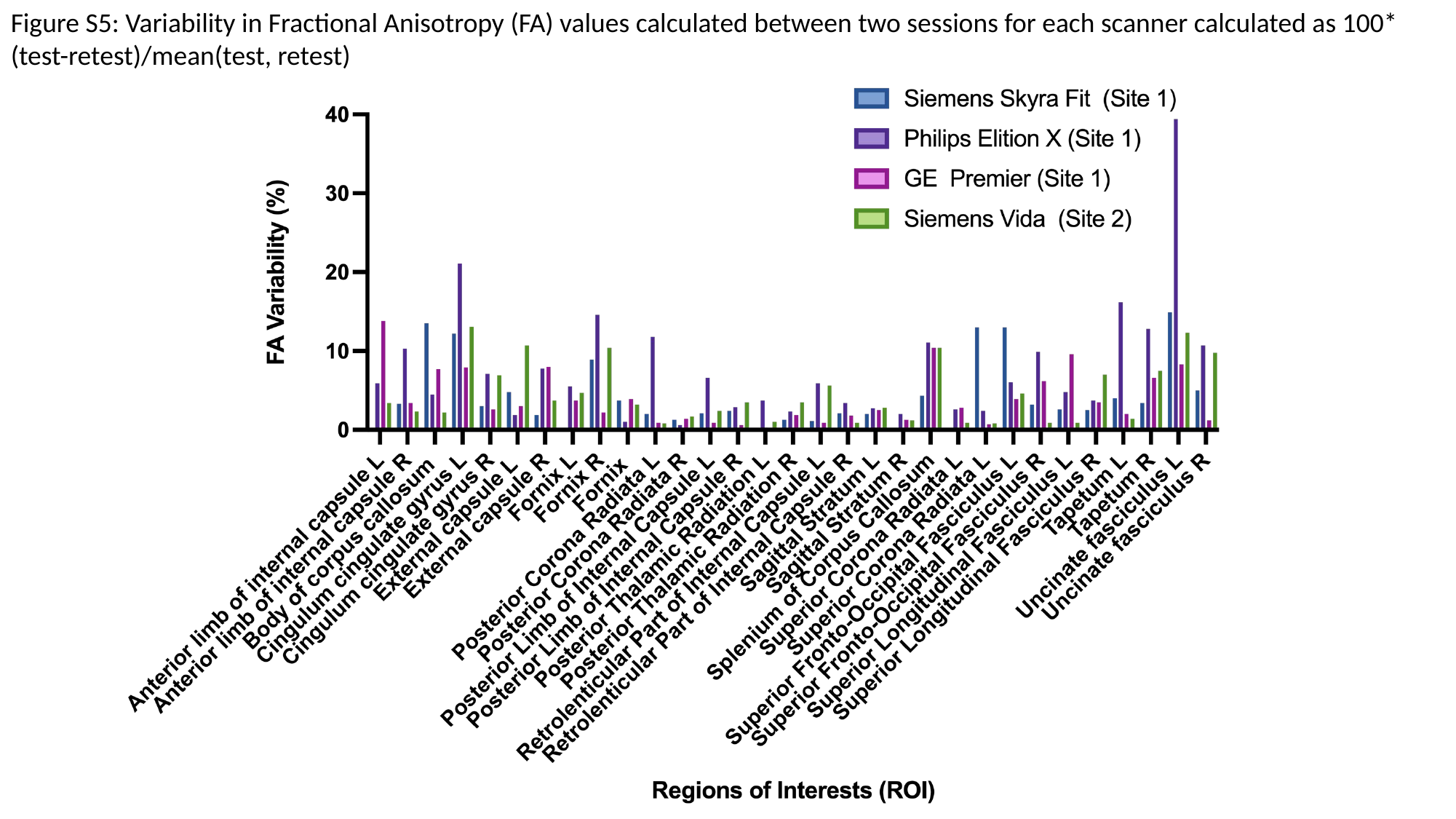

Figure S5: Variability in Fractional Anisotropy (FA) values calculated between two sessions for each scanner calculated as 100* (test-retest)/mean(test, retest)
